# Intra-Fraction Motion Management for Radiosurgical Treatments of Trigeminal Neuralgia: Clinical Experience, Imaging Frequency, and Motion Analysis

**DOI:** 10.7759/cureus.14616

**Published:** 2021-04-21

**Authors:** Nzhde Agazaryan, Stephen Tenn, Nader Pouratian, Tania Kaprealian

**Affiliations:** 1 Radiation Oncology, David Geffen School of Medicine at University of California, Los Angeles (UCLA), Los Angeles, USA; 2 Neurosurgery, David Geffen School of Medicine at University of California, Los Angeles (UCLA), Los Angeles, USA

**Keywords:** radiosurgery, trigeminal, neuralgia, imaging, motion

## Abstract

Purpose

The aim of this study is to evaluate the patient positioning and intra-fraction motion management performance of an image-guidance protocol established for radiosurgical treatments of trigeminal neuralgia patients. Specifically, it also aims to analyze patient motion data for the evaluation of current motion tolerance levels and imaging frequency utilized for repositioning patients.

Methods

A linear accelerator equipped with ExacTrac is used for patient positioning with stereoscopic imaging and treatments. Treatments are delivered with 4-mm conical collimators using seven equally spaced arcs. Arcs are 20 degrees apart and span 100 arc degrees each. Following initial ExacTrac positioning, cone beam computed tomography (CBCT) is obtained for independent confirmation of patient position. Patients are then stereoscopically imaged prior to the delivery of each arc and repositioned when 0.5-mm translational tolerance in any direction is exceeded. After the patient has been repositioned, verification stereoscopic images are obtained. Data from 48 patients with 607 image pairs were analyzed for this study.

Results

Over the course of 48 patient treatments, the mean magnitude of mean 3D deviations was 0.64 mm ± 0.12 mm (range: 0.07-2.74 mm). With the current 0.50-mm tolerance level for repositioning, patients exceeded the tolerance 51.4% of the time considering only images following an arc segment. For those instances, patients were repositioned with a mean magnitude of 0.85 mm ± 0.15 mm (1 SD). For a 0.25-mm tolerance level, 86.1% of arc segments would have required repositioning following the delivery of an arc segment, with a mean magnitude of 0.68 mm ± 0.12 mm. Conversely, for 0.75-mm and 1.00-mm tolerance levels, the tolerance would have been exceeded only 21.5% and 6.6% of instances following the delivery of an arc segment, with a mean magnitude of 1.08 mm ± 0.21 mm and 1.34 mm ± 0.24 mm, respectively. Each repositioning adds approximately 2 minutes to treatment time, which accounts for parts of the variability in patient treatment times. Following the initial ExacTrac and CBCT, the mean treatment time from first arc to treatment end was 57 minutes (range: 33-63 minutes).

Discussions

The current 0.50-mm tolerance level results in a clinically manageable but significant number of patient repositions during trigeminal neuralgia treatments. Frequent patient repositioning can result from actual patient motion convolved with the accuracy and precision limitations of the image analysis. Increasing the repositioning tolerance could more selectively correct for actual patient motion and shorten the treatment time at the expense of more variations in patient position. A more lenient tolerance level of 0.75 mm would decrease the repositioning rate by approximately a factor of 2; however, the permissible magnitude of motion will increase, leading to possible dosimetric consequences. Once treatment begins, there was no trend as to when patients exceeded the tolerance.

Conclusions

Current imaging protocol for patient positioning and intra-fraction motion management fits the clinical workflow with clinically acceptable residual patient motion. The next important step would be to assess how the number of repositions and magnitude of residual movements affect treatment outcomes.

## Introduction

Trigeminal neuralgia (TN) is a chronic pain condition of the trigeminal nerve that affects approximately five out of 100,000 persons in the United States [1]. Primary treatment options include pharmacological and surgical interventions [2]. For some patients who are refractory to drugs and for whom surgical intervention is contraindicated, stereotactic radiosurgery (SRS) is an effective treatment option [3].

Both frame and frameless SRS techniques have been utilized to successfully treat TN [4]. With frame-based methods, immobilization and localization are both accomplished via a rigid frame that is secured via screws embedded into the patient's skull. With frameless treatment techniques, the patients are typically immobilized with a thermoplastic mask system and localization is accomplished separately via image guidance. Unlike frame-based systems, patients are still able to move within thermoplastic masks [5]. To compensate for this intra-fraction movement, image-guided radiation therapy is employed during the treatment to detect and compensate for the patient’s position.

Some image guidance systems utilize a technique where the treatment beams are adjusted to coincide with the updated target position for any displacements that are detected [6]. This is typical of the CyberKnife system, for example. For other systems, the patient must be relocated back to the machine’s isocenter via couch movements. This latter method is typically associated with gantry-mounted treatment units. Because corrections are applied by moving the patient, users will typically select a threshold value for the allowed deviation. Displacements larger than the threshold value would require the patient’s position to be corrected and displacement within the threshold would be ignored. Many institutions will also require verification imaging whenever displacements are corrected. This is because the action of moving the couch can cause the patient to move relative to the couch, reducing the overall repositioning accuracy. Thus, verification imaging ensures that the patient is correctly aligned before treatment proceeds. While there are many reports in the literature discussing both frameless localization accuracy and intra-fraction motion [5-8], there has not been a detailed study of the correction threshold value used and its effect on treatment time and overall treatment uncertainty. The aim of the current study is to analyze the position data obtained from our TN treatments and evaluate the threshold value used for repositioning with respect to alignment uncertainty and positional correction frequency. Part of this study started in 2013, and some of the results of this study were presented at the International Stereotactic Radiosurgery Society, American Association of Physicists in Medicine, and other national and international conferences.

## Materials and methods

**Figure 1:**
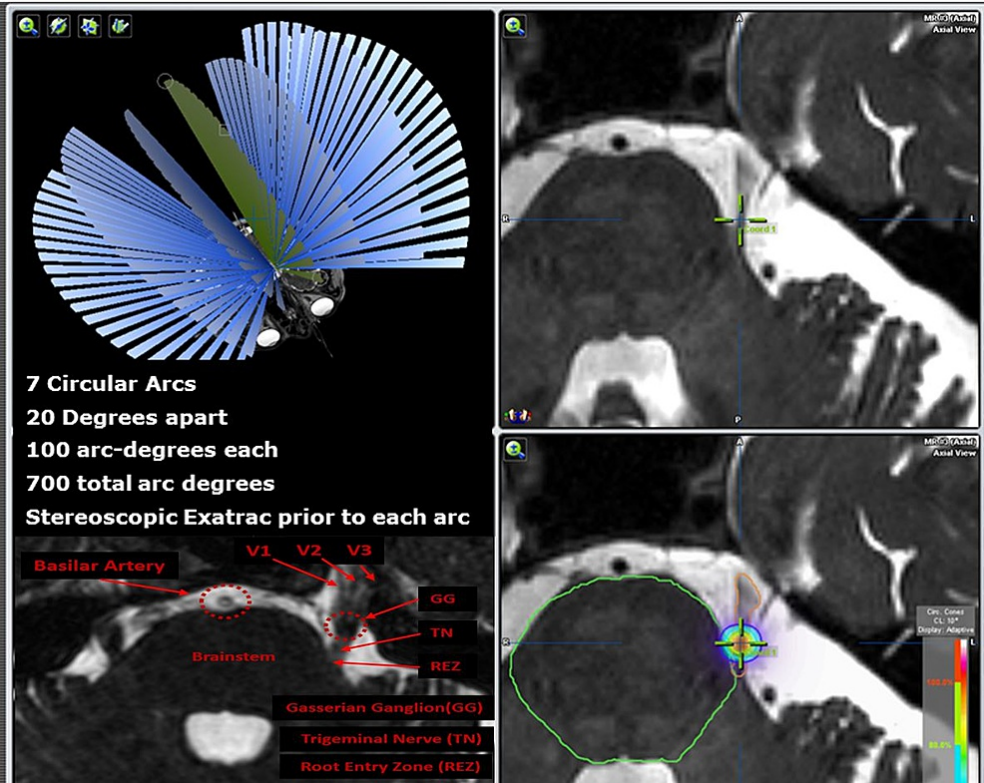
Treatments are delivered to the root entry zone with a 4-mm collimator using seven equally spaced arcs. Arcs are 20 degrees apart and span 100 arc degrees each. Prescribed radiation dose to the maximum dose point is 90 Gy.

TrueBeam STx and Novalis Tx (Varian Medical Systems, Palo Alto, CA, and Brainlab, Munich, Germany) equipped with identical ExacTrac 6DOF stereoscopic kV imaging (ExacTrac 6.2, Brainlab) are currently used in our department for patient positioning with stereoscopic imaging and treatments. Treatments are delivered with 4-mm conical collimators using seven equally spaced arcs. Arcs are 20 degrees apart and span 100 arc degrees each (Figure 1). Data from 48 patients with 607 image pairs were retrospectively analyzed for this study.

The patients’ simulations include images from Siemens CT scanner (Sensation Open, Siemens Healthcare, Erlangen, Germany) with 1.5-mm slice thickness. The patients included in this study were immobilized with thermoplastic mask system (Brainlab). MRI image sets include 1-mm isotropic MPRAGE T1 with contrast administration and 1-mm constructive interference in steady state (CISS) T2-weighted MRI. The CISS scan is a heavily T2-weighted scan that generates a large contrast between cerebrospinal fluid (CSF) in the prepontine cistern and the brainstem and nerves. This scan is typically used to define the target and guide placement of the isocenter for treatment delivery. The fusion evaluation includes the cochlear structures on the side ipsilateral to the target nerve since this structure can be easily visualized on both scans. In cases where the patient cannot undergo MRI, the alternative is to obtain a CT cisternogram.

iPlan RT (Brainlab) is used for planning these cases. Structures at risk are contoured on the MR images. The isocenter and the dose distribution is positioned such that the root entry zone of the trigeminal nerve is covered with the 50% isodose line just abutting the brainstem. The plan is normalized to the maximum dose point and the maximum dose point is 90 Gy in a single fraction. The brainstem volume receiving 12 Gy is kept less than 0.3 cc. The volume for a point constraint is specified as 0.03 cm^3^ in RTOG (Radiation Therapy Oncology Group) 0631. This is more restrictive than the 0.03 cm^3^ definition of a point in TG (Task Group) 101. The optic nerve, chiasm, and cochlea doses are limited to 8 Gy with the same 0.03 cm^3^ definition, and the lens point doses is limited to 4 Gy.

Prior to treatment with stereotactic conical collimator, a pre-treatment modified Winston-Lutz test (cardinal angles only) and ExacTrac verification test are performed [9]. The test is performed with a 7.5-mm conical collimator and a 5-mm radiopaque pointer. This test is repeated any time the collimator mount for the stereotactic conical collimators is put in place. The 4-mm conical collimator Winston-Lutz test is not performed prior to each patient treatment; however, it can be periodically checked using the method described later in this section.

**Figure 2:**
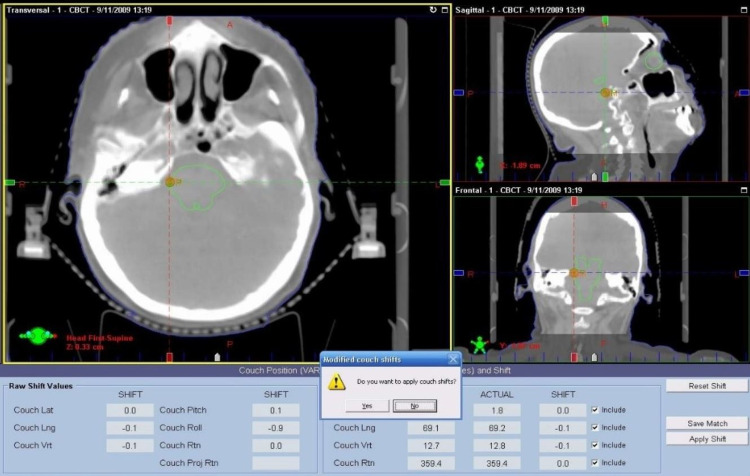
Following initial ExacTrac positioning, CBCT is obtained for independent confirmation of patient position. The patient is then stereoscopically imaged prior to the delivery of each arc and repositioned when 0.5-mm translational tolerance in any direction is exceeded. CBCT, cone beam computed tomography

Trigeminal SRS is one of the high-risk procedures performed in the department. To ensure the target alignment is correct, we perform cone beam computed tomography (CBCT) following the initial ExacTrac setup and prior to the first beam being delivered (Figure 2). The purpose of the CBCT is not to adjust alignment of the patient, but it is simply another verification method that has been calibrated independent of ExacTrac. Deviations in translations up to 2 mm are considered normal, and treatment may proceed. Any deviations in patient positioning detected by the CBCT that are larger than 2 mm is not be used to shift the patient. Instead, the deviations must be reviewed and investigated by the physicist supervising the delivery.

In terms of patient positioning, following initial ExacTrac positioning, CBCT is obtained for independent confirmation of patient position. The patient is then stereoscopically imaged prior to the delivery of each arc and repositioned when 0.5-mm translational tolerance in any direction is exceeded. After the patient has been repositioned, verification stereoscopic images are obtained. The treatments are managed with ARIA® Record and Verify (RV) System (ARIA 13.6, Varian Medical Systems, Palo Alto, CA).

For TN treatments, we do not make robotic rotational corrections around the lateral (pitch) or longitudinal (roll) patient axes for deviations under 3 degrees. While the ExacTrac system can make these corrections automatically, the addition of these corrections adds complexity and increased risk of collisions between the couch top and the conical collimator without dosimetric improvement. Rotational corrections around the lateral axis have a pivot point near the patients' feet when using the Brainlab Robotic Couchtop. This means that for each degree of rotational correction, there is approximately 3 cm of vertical couch translation at the patient's head. therefore, while rotational corrections may be performed to sub-degree accuracy, the couch translations that accompany these corrections were concerning to us for this treatment. Additionally, the conical collimators can collide with the Brainlab Imaging Couchtop Frameless Extension, depending on the location of the isocenter with respect to the couch. As part of our quality assurance process, we test for collisions following plan approval to ensure that there will be no issues on the day of treatment. However, pitch and roll uncertainty are not included in this testing. Due to these considerations, we have elected to treat TN without enabling automatic rotational corrections.

**Figure 3:**
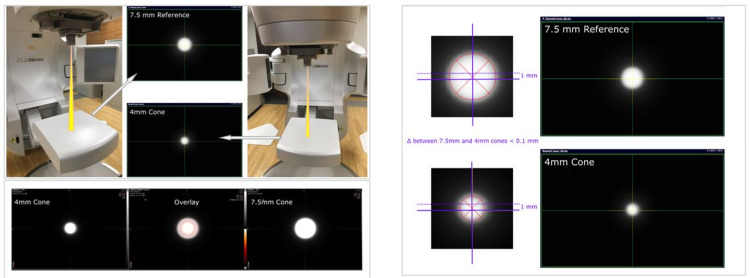
EPID-based conical collimator positioning accuracy test for the 4-mm conical collimator. The positioning of the 4-mm cone is evaluated against the positioning of the 7.5-mm cone. The 7.5-mm cone itself is tested prior to this with a Winston-Lutz test using a 5-mm radiopaque pointer. EPID, electronic portal imaging device

As we have discussed, prior to any conical collimator treatment, a pre-treatment Winston-Lutz test (cardinal angles only) and ExacTrac verification test are performed with a 7.5-mm conical collimator and a 5-mm radiopaque pointer. A commonly encountered challenge is positioning tests for the 4-mm or 5-mm conical collimators, which are not performed per-patient basis. For that purpose, EPID (electronic portal imaging device) based conical collimator positioning accuracy test is employed for the 4-mm and 5-mm conical collimator positioning accuracy (Figure 3). The positioning of the 4-mm and 5-mm cones are evaluated against the positioning of the 7.5-mm cone. With a cone mount in place, images with 7.5-mm and 4-mm cones are obtained and overlaid for evaluation. In this example, the discrepancy between the 7.5-mm image center and 4-mm image center is less than 0.1 mm. Prior to this comparative test, the 7.5-mm cone itself is tested with a Winston-Lutz test. The Winston-Lutz test is performed with a 7.5-mm conical collimator and a 5-mm radiopaque pointer. This Winston-Lutz test is repeated any time the collimator mount for the stereotactic conical collimators is put in place prior to patient treatments. The 4-mm conical collimator test described is not performed prior to each patient treatment; however, it should be periodically checked.

## Results

Over the course of 48 patient treatments, the mean magnitude of mean 3D deviations was 0.64 mm ± 0.12 mm (range: 0.07-2.74 mm). With the current 0.50-mm tolerance level for repositioning, patients exceeded the tolerance 51.4% of the time considering only images following an arc segment. For those instances, patients were repositioned with a mean magnitude of 0.85 mm ± 0.15 mm (1 SD).

**Figure 4:**
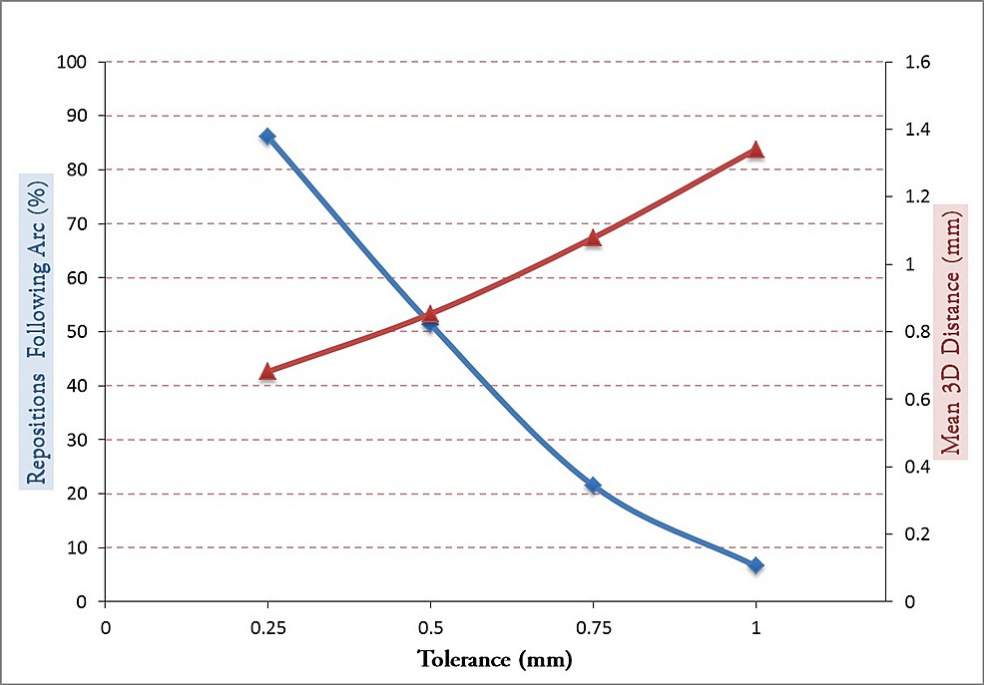
With the current 0.50-mm tolerance level for repositioning, patients exceeded the tolerance 51.4% of the time considering only images following an arc delivery. For those instances, patients were repositioned with a mean magnitude of 0.85 mm. With 0.75-mm and 1.00-mm tolerance limits, the tolerance would have been exceeded only 21.5% and 6.6% of the time, respectively.

For a 0.25-mm tolerance level, 86.1% of arc segments would have required repositioning following the delivery of an arc segment, with a mean magnitude of 0.68 mm ± 0.12 mm (Figure 4). Conversely, for 0.75-mm and 1.00-mm tolerance levels, the tolerance would have been exceeded only 21.5% and 6.6% of instances following the delivery of an arc segment, with a mean magnitude of 1.08 mm ± 0.21 mm and 1.34 mm ± 0.24 mm, respectively.

Each repositioning adds approximately 2 minutes to treatment time, which accounts for part of the variability in patient treatment times. Following the initial ExacTrac and CBCT, the mean treatment time from first arc to treatment end was 57 minutes (range: 33-63 minutes).

## Discussion

Patient motion management is critical during frameless TN treatments with a radiosurgical approach. Frameless treatments with a thermoplastic mask provide patient comfort and the patients do not need to be treated the day of the simulation, allowing more time for treatment planning and quality assurance. However, frameless treatments potentially allow for more patient motion than the framed-based approach. This potential motion issue is addressed by intra-fraction monitoring and repositioning with image guidance. Studies show clinically comparable or better results with frameless image-guided versus framed radiosurgery systems in terms of setup accuracy and intra-fraction motion [10-11]. For example, it was reported that the overall system accuracy with a thermoplastic mask and stereoscopic image-guided system is comparable to a framed method [10]. However, another study reported that the intra-fraction motion is larger with thermoplastic mask immobilization, but it can remain within a clinically acceptable range with appropriate image guidance [11]. Our work is focused on this aspect of the patient treatment, where tolerance levels, repositioning rates, and residual movements are investigated.

The results of this study show that the current 0.50-mm tolerance level results in a clinically manageable but significant number of patient repositions during TN treatments. Patient repositioning can result from actual patient motion convolved with the accuracy and precision limitations of the image analysis. Hence, not all repositions are triggered merely because the actual patient motion alone exceeded the 0.50-mm tolerance level. Often it is the combination of the patient motion and variability in the system’s accuracy to determine the patient position. With 0.50-mm tolerance level, the mean treatment time from first arc to treatment end is 57 minutes. Increasing the repositioning tolerance could more selectively correct for actual patient motion and shorten the treatment time at the expense of more variations in patient position. A more lenient tolerance level of 0.75 mm would decrease the repositioning rate by approximately a factor of 2; however, the permissible magnitude of motion will increase, leading to possible dosimetric consequences.

In this study, we attempted to observe patterns in patient motion; however, once the patient treatments begin, there were no trends as to when patients exceeded the tolerance. Hence, intra-fraction motion management at regular intervals is critical. In this study, we observed patient motion and correct alignment prior to each arc. Other studies with a capability of continuous monitoring have reported that a small population of patients may exhibit occasional large transient movement even under well-controlled conditions, where the movement appears random and transient, returning back to a position closer to the initial setup location [12]. Ultimately, while the clinical advantage of being able to continuously monitor patient position needs to be proven, it can only help the treatment outcome, if done without significantly increasing treatment times.

## Conclusions

Our current frameless trigeminal radiosurgery protocol for patient positioning and intra-fraction motion management is based on ExacTrac kV stereoscopic imaging. We image prior to each arc and correct any translational deviations exceeding 0.5 mm per axis. This tolerance value facilitates an efficient workflow with clinically acceptable residual position uncertainty during treatment.

Additionally, immediately following the initial stereoscopic positioning, we verify the patient setup using CBCT. Including CBCT verification extends overall treatment time by 3-5 minutes. Excluding the initial ExacTrac and CBCT imaging time, the mean time from first beam delivery to completion of treatment is 57 minutes, which is clinically acceptable for the treatment of TN.

This study will be expanded to assess how patient motion during the treatment affects treatment outcome. The next phase of this study will assess the impact repositioning frequency and magnitude of residual movements have on treatment outcomes.
